# Increased Risk of Cryptogenic Stroke Associated with Patent Foramen Ovale in Young Adults

**DOI:** 10.7759/cureus.53502

**Published:** 2024-02-03

**Authors:** Alec J Knupp, Douglas A Smith

**Affiliations:** 1 Medical School, Lake Erie College of Osteopathic Medicine-Bradenton College of Osteopathic Medicine, Bradenton, USA; 2 Emergency Medicine, Nuvance Health, Danbury Hospital, Danbury, USA

**Keywords:** medical icu, icu, middle cerebral artery (mca), young adult ischemic stroke, paradoxical cerebral embolism, cryptogenic stroke, patent foramen ovale (pfo)

## Abstract

Ischemic stroke is defined as a reduction in blood flow to brain tissue that results in the deterioration and death of neurons in a matter of minutes. While often seen in older patients with a history of atherosclerosis of the major arteries, a subset of ischemic strokes occur in younger individuals with minimal to no prior risk factors. Further evaluation of these unknown, or cryptogenic, strokes has yielded positive findings of a patent foramen ovale (PFO) in a concerning number of cases. Cryptogenic strokes attributable to PFO present an important clinical occurrence because they do not fit the typical template regarding those most at risk for such acutely devastating outcomes, making their identification uniquely important for both immediate and long-term patient care.

A 20-year-old Hispanic female presented to the emergency department for evaluation of neurological symptoms indicating obstruction of a major cerebral vessel. After being placed on stroke alert and found to have an embolus occluding the left middle cerebral artery (MCA) via non-contrast computed tomography (CT), tissue plasminogen activator (tPA) was administered, and mechanical thrombectomy was performed to restore blood flow. Following stabilization, further testing done on the patient revealed a substantial PFO that likely allowed for the crossing of an embolus from venous blood returning to the heart directly into the arterial circulation. The patient opted for cardiac monitor placement and has remained asymptomatic to this point while awaiting surgical repair.

This case demonstrates an unusual presentation of ischemic stroke in a young individual with no reported risk factors and highlights the importance of screening for large PFO in patients prior to a serious cerebrovascular accident. It is our hope that highlighting this case may heighten awareness of this condition and allow for timely recognition from medical personnel who may encounter this same medical emergency in the future.

## Introduction

Patent foramen ovale (PFO) is a congenital cardiac defect characterized by an unfused area of tissue between the right and left atria. Patent foramen ovale is required during fetal development for the passage of oxygenated blood from the right atria into the left atria, directly entering systemic circulation while avoiding pulmonary circulation [1]. Normally, this gap becomes fused shortly after birth when pulmonary and systemic vascular resistance decreases and increases, respectively. Despite PFO being present in 25%-30% of the population and most cases being asymptomatic, the condition represents a concerning risk factor for potentially fatal conditions, among which is cryptogenic stroke [1].

Cryptogenic stroke is defined as a cerebral occlusion unattributable to cardio-embolism or large artery atherosclerosis [2]. Patent foramen ovale has been associated with stroke because of the unfiltered access of venous thrombi to the cerebral vasculature of the brain. The significance is rooted in the sheer ease of entry for clots to pass directly from the right atria into the left atria and then systemic circulation coined the paradoxical embolus. An estimated 40% of strokes in patients under the age of 55 are cryptogenic in nature [3]. A prospective PFO-atrial septal aneurysm (ASA) study found that in 581 patients (mean age: 42), 37% were diagnosed with PFO [4]. These data reveal that with younger age and thus, a decreased likelihood of prevalent risk factors seen in older patients (progressed atherosclerosis), PFO may represent a significant predisposition to unexpected stroke. The scale of risk further falls on the anatomical characteristics of each individual PFO as well as surrounding conditions of genetic hypercoagulability, medications, or vascular trauma [5, 6].

The size and shape of an identified PFO are of primary clinical importance and have been studied and determined to play a role in the likelihood of cerebral occlusion. The presence of larger PFOs (defined as over 2 mm of separation between the septum primum and septum secundum) has yielded more significant findings on brain autopsy than smaller separations [7, 8]. Identification of PFOs with an assessment of risk based on size is, at present, the only preventative measure.

The purpose of presenting this case is to bring attention to an asymptomatic congenital condition that has serious potential consequences for undiagnosed patients.

## Case presentation

A 20-year-old Hispanic female with no previous stroke risk factors reported to the emergency department for urgent evaluation of sudden onset right-sided facial droop, expressive aphasia, and bilateral loss of upper extremity motor coordination. She stated that the onset began at 1800 hours when she noticed an impaired ability to use her computer keyboard, followed by the ability to smile only with the left side of her mouth. Upon initial evaluation at the emergency department, her symptoms had resolved entirely with a National Institutes of Health (NIH) stroke scale score of 0 at 1952 hours. Her review of the systems returned negative for any indication of an impending stroke prior to the episode. She denied any recent visual problems, fever, chest pain, syncope, or unusual skin changes. There was no social history of smoking, family history of a cerebral vascular accident, or oral contraceptive use. There was also no report of recent surgery. Her initial ophthalmoscopic exam revealed intact extraocular muscles and pupils bilaterally reactive to light with no afferent pupillary defect. The cardiac exam was negative for any murmur on auscultation, and the neurologic exam showed that cranial nerves (CN) II-XII were intact with facial symmetry and orientation x3. The musculoskeletal exam was negative for calf swelling. Laboratory testing returned normal, besides elevated white blood cells (WBC). Lab results for blood chemistry and urinalysis were within the normal range. Coagulation studies revealed a slightly elevated prothrombin time (PT) and partial thromboplastin time (PTT); however, the international normalized ratio (INR) was within range (Table 1).

**Table 1:** Initial laboratory testing UA: urinalysis; RBC: red blood cells; WBC: white blood cells; BUN: blood urea nitrogen; PT: prothrombin time; PTT: partial prothrombin time; INR: international normalized ratio; coag: coagulation profile; hCG: human chorionic gonadotropin

Investigation name	Patient value	Reference range
Urinalysis	---	---
UA pH	6.5	4.5-8
UA specific gravity	1.003	1.005-1.025
UA glucose	None	≤130 mg/dL
UA ketones	None	None
UA protein	Negative	≤150 mg/dL
UA RBC	0-2	≤2 RBCs/hpf
UA bacteria	53 / uL	None
UA WBC	0-2	≤2-5 WBCs/hpf
UA nitrite	Negative	Negative
UA leukocyte esterase	Negative	Negative
UA hyaline casts	0-8	0-5 hyaline casts/lpf
UA-amphetamines	Negative	Negative
UA-cocaine	Negative	Negative
UA-opiate	Negative	Negative
Chemistry	---	---
Sodium	142 mmol/L	135 to 145 mmol/L
Potassium	3.6 mmol/L	3.5 to 5.20 mmol/L
Chloride	105 mmol/L	96 to 106 mmol/L
Bicarbonate (HCO3)	25 mmol/L	18-28 mmol/L
Anion gap	12 mmol/L	≤12 mmol/L
BUN	9 mg/dL	6 to 20 mg/dL
Creatinine	0.6 mg/dL	0.6 to 1.3 mg/dL
Glucose	108 mg/dL	70 to 100 mg/dL
Coag/D-Dimer	---	---
PT	12.3 sec	9.1−12.0 sec
PTT	33.9 sec	24−33 sec
INR (ratio)	0.9	0.9−1.2
UA/Serum pregnancy	---	---
Beta-hCG	negative	negative

The INR is a measure used to assess the body’s ability to form clots in an appropriate amount of time, with a lower value indicating a more probable state of hypercoagulability [9].

**Figure 1:**
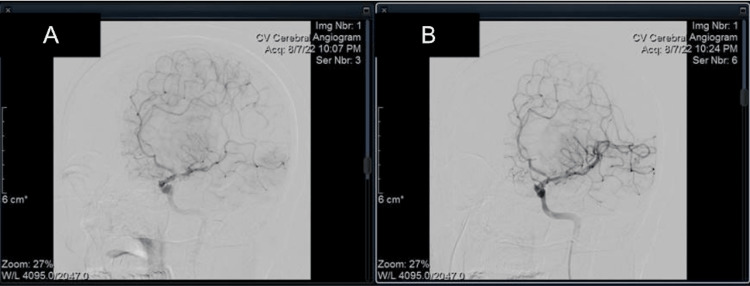
Cerebral angiogram (A) Imaging of the left internal carotid artery shows identifiable occlusion of the proximal left posterior division of the middle cerebral artery with impairment of cerebral blood flow prior to thrombectomy; (B) Post mechanical thrombectomy, blood flow through the previously occluded vessel is restored with no residual blockage.

Despite a normal presentation at the time of evaluation, the patient was placed on a stroke pathway given the concerning symptoms reported. Other differential diagnoses were considered, including complex migraine, idiopathic cerebral vasospasm, and focal seizures. A stroke alert was not felt to be indicated after consultation with neurology, which determined a transient ischemic attack (TIA) to be the likely cause of presentation. However, the patient went for non-contrast CT imaging followed by a CT angiogram, and shortly after returning to her room, she suffered another episode of expressive aphasia, which prompted a level 1 stroke alert. At this time, neuroradiology notified the attending physician that an occlusion was present in the proximal, posterior division of the left middle cerebral artery (MCA). A cerebral angiogram confirmed the vessel blockage while ruling out any dissection or vasculitis (Figure 1A).

The remaining large arteries were patent, with no occlusion or hemodynamically significant stenosis within the cervical carotid or vertebral arteries.

The patient was administered a tissue plasminogen activator (tPA) and taken to the catheterization lab for an immediate left MCA mechanical thrombectomy. Following successful re-perfusion of the affected artery, there was no return of symptoms (Figure 1B).

Following stabilization, further workup revealed a moderately patent foramen ovale with a positive bubble study per transthoracic echocardiography (TTE). The tunnel of the PFO measured 5 mm, with a positive septal aneurysm and an excursion distance of 10 mm. Color flow Doppler confirmed a right-to-left shunt across the PFO. Lower extremity duplex and magnetic resonance venography (MRV) of the pelvis ruled out any active deep vein thrombosis (DVT). Antibody testing for autoimmune-linked anti-nuclear antibody, centromere antibody, anti-dsDNA, anti-SCL 70, anti-SSA/SSB, anti-cardiolipin, and rheumatoid factor all returned negative (Table 2) [10].

**Table 2:** Antibody serology testing

Antibody	Associated condition
Anti-nuclear antibody (ANA)	Non-specific; systemic lupus erythematosus (SLE), rheumatoid arthritis, and Sjögren’s syndrome
Centromere antibody	Limited scleroderma
Anti-dsDNA	Systemic lupus erythematosus
Anti-cardiolipin	Systemic lupus erythematosus and antiphospholipid syndrome
Anti-SCL 70	Diffuse scleroderma
Anti-SSA/SSB	Sjögren’s syndrome
Rheumatoid factor	Rheumatoid arthritis

The patient was started on aspirin 81 mg and atorvastatin 80 mg daily therapy. She opted for the noninvasive placement of a cardiac monitor for one month. The patient was asymptomatic as of the most recent follow-up.

## Discussion

Of all deaths in the United States, one in 20 is a result of a stroke. Of this 5% of all deaths, 87% are ischemic in nature [9]. Whether fatal or not, 10%-14% of all ischemic strokes occur in young individuals, and cryptogenic strokes account for 40% of this [10]. With PFO being a primary risk factor for cryptogenic stroke in young adults, these numbers help place into perspective the importance of recognizing and exploring both primary and secondary prevention of cerebrovascular accidents resulting from this condition.

This case presented a young patient with no identified risk factors who presented with an acute ischemic stroke, most likely as a result of a paradoxical embolism traveling through an undiagnosed PFO. The initially normal clinical evaluation of this patient after reporting neurological deficits prior to arrival demonstrated a transient restoration of blood flow to her brain parenchyma. The physician’s decision to have non-contrast CT imaging done is currently the primary recommendation from the American Heart Association (AHA) for an initial evaluation of a possible stroke [11]. Of note, non-contrast scanning is preferred over the use of contrast because of time constraints as well as interference with the detection of hemorrhage that contrast dye may contribute [12]. Guidelines for CT interpretation of cerebral stroke include three stages: acute (under 24 hours), subacute (24 hours to five days), and chronic (weeks) [11]. Occlusion of the proximal MCA results in increased density on imaging, or hyperattenuation, and can be seen in the acute stage of stroke. In Figure 1A, clear hyperattenuation can be seen in the region of the patient’s proximal, posterior MCA. The advantages of non-contrast CT imaging in stroke presentation are that it can rule out intracerebral hemorrhage and can be performed in 60 seconds [11]. This case highlights the importance of using CT to evaluate cerebral vasculature, even in an unusually presenting demographic with alleviated symptoms.

Important results in the patient’s evaluation prior to diagnosis of a PFO were a lack of calf swelling on physical examination and negative autoantibody titer results on serology testing (Table 2). Calf swelling is seen in acute DVT and is a potent source of breakaway emboli [13]. As previously mentioned, emboli from a DVT can result in an acute stroke when a PFO is present. Autoantibody testing is critical to perform when diagnosing the etiology of a clot because certain autoimmune conditions, particularly systemic lupus erythematosus (SLE) and anti-phospholipid syndrome, may lead to hypercoagulable states [14].

An interesting co-finding was an ASA, defined as a congenital bulging of the atrial septum near the fossa ovalis, the remnant of the fetal foramen ovale [8]. Coexistence with an ASA is rarer than that with a sole PFO, and the presence of both can accentuate the diameter of the shunt. Identifying the patient’s PFO along with ASA was done through a transesophageal echocardiogram (TEE) and is currently the gold standard [8]. Treating an identified PFO by closing the open space of tissue is done through catheterization, which is the primary minimally invasive surgical solution. However, it comes with the risks of embolization and procedure-related structural damage [15]. Anticoagulant medications such as warfarin have been suggested as secondary prevention following a cryptogenic stroke, but a definitive ‘best anticoagulant’ is still controversial [9]. With this, a meta-analysis of three randomized controlled trials of PFO management in patients who have experienced at least one cryptogenic stroke showed that shunt closure is the preferred treatment over pharmacological prevention following cryptogenic stroke with PFO [9].

**Figure 2:**
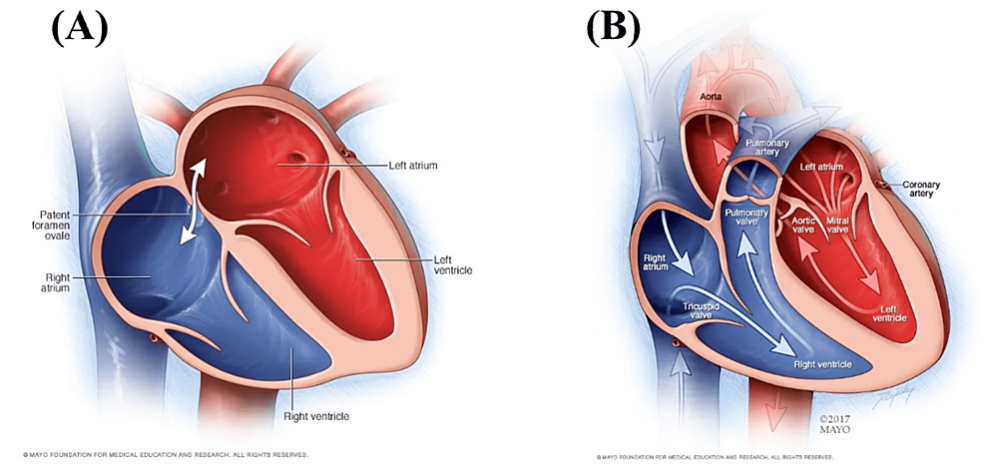
Fetal and adult cardiac structure (A) Patent foramen ovale seen during normal fetal development; (B) Normal cardiac structure and blood flow in adults with fused fossa ovalis. Permission for the use of the figures has been granted by Mayo Clinic [16].

Studies and meta-analyses continue to be published on the diagnosis and treatment of PFO and cryptogenic stroke. The utility of this case report is to not only blueprint findings but also place them in the context of a single patient. Specifically for current and future emergency medicine providers, taking the knowledge on a topic of unusual stroke and placing it within the presentation of a real patient can serve as an invaluable frame of reference for future clinical decision-making. Treatment of acute ischemic stroke is well-established regardless of cause; however, the second leg of a successful outcome for a patient with a PFO is what occurs after they are stabilized. If an embolism occurs once, there is a risk of a second occurrence if no intervention is performed. The emphasis of this case begins with instilling in practice routinely ruling PFO in or out for every patient whose stroke etiology is not definitive. With this, sample size is a limitation and should be considered when applying this case report to any given patient with PFO. Presentations vary, and one may have fewer or more symptoms compared to the patient discussed. In regard to treatment following diagnosis, the recorded size of this patient’s PFO (5 mm) complements the literature stating increasing risk past 2 mm in diameter warrants consideration for immediate management or closure. Figure 2A displays blood flow through the appropriately open foramen ovale in a fetal heart, while Figure 2B shows flow through an adult with the proper fusing of the foramen ovale into the fossa ovalis [16]. 

## Conclusions

This case presents a minimally predictable yet highly threatening cerebral occlusion by a mechanism of paradoxical embolism through a previously undiagnosed PFO. The nature of the patient’s presentation demonstrates that without proper diagnosis and prophylactic management of clinically defined 'moderate-to-large' (over 2 mm of septal separation) PFO, the incidence of cryptogenic strokes can materialize in a young demographic. The patient’s lack of risk factors such as oral contraceptive use, smoking, or family history highlights the importance of finding significantly-sized PFOs in all patients prior to an event such as that experienced by this patient.

Heightened awareness of the possibility of clinically significant PFO in any given newborn or young adult will advance a more consistent practice of primary intervention in cases such as this. Basic provider knowledge, such as the PFO murmur characteristic, allows for inexpensive initial screening. However, reliance on auscultation of a systolic ejection murmur localized to the pulmonic valve, characteristic of a PFO, has sensitivity limitations. Transesophageal echocardiography and saline contrast (bubble) studies serve as the primary means of diagnosis should suspicion warrant further work-up. The standing concern is broad access to these more specific evaluations for every person in the United States.

This case serves as a cautionary tale of an often asymptomatic but potentially lethal congenital condition that lies dormant in many individuals. This patient had access to medical care immediately, which presents the hypothetical ponderance of another outcome if she had not. The clinical point of reporting this case is to bring awareness to an incredibly dangerous yet often unrecognized risk factor in a substantial number of young people.
